# A Pool of Bacterium-like Particles Displaying African Swine Fever Virus Antigens Induces Both Humoral and Cellular Immune Responses in Pigs

**DOI:** 10.3390/vaccines13010005

**Published:** 2024-12-24

**Authors:** Jingshan Huang, Hongxia Wu, Tianqi Gao, Huanjie Zhai, Assad Moon, Xin Song, Shuwen Li, Zhanhao Lu, Jing Lan, Dailang Zhong, Xinyu Zhang, Hua-Ji Qiu, Yongfeng Li, Yuan Sun

**Affiliations:** 1 State Key Laboratory for Animal Disease Control and Prevention, National African Swine Fever Para-Reference Laboratory, Harbin Veterinary Research Institute, Chinese Academy of Agricultural Sciences, Harbin 150069, China; hjs45333@163.com (J.H.); wuhongxia@caas.cn (H.W.); 19990736668@163.com (T.G.); 15237372162@163.com (H.Z.); 2021y90100029@caas.cn (A.M.); songxinor@163.com (X.S.); lishuwen176@163.com (S.L.); luzhanhao106@163.com (Z.L.); lanjing121@163.com (J.L.); hsrzlxcrb2021zdl@163.com (D.Z.); 15978702290@163.com (X.Z.); qiuhuaji@caas.cn (H.-J.Q.); 2 College of Animal Science and Technology, Jilin Provincial Engineering Research Center of Animal Probiotics, Key Laboratory of Animal Production and Product Quality Safety of Ministry of Education, Jilin Agricultural University, Changchun 130118, China

**Keywords:** bacterium-like particles, African swine fever, Gram-positive enhancer matrix, immunological evaluation

## Abstract

Background/Objectives: African swine fever (ASF), caused by African swine fever virus (ASFV), poses a significant threat to the global swine industry. This underscores the urgent need for safe and effective ASF vaccines. Methods: Here, we constructed five bacterium-like particles (BLPs) that each display one of the five ASFV antigens (F317L, H171R, D117L, B602L, and p54) based on the Gram-positive enhancer matrix-protein anchor (GEM-PA) system. GEM is a bacterial particle that contains only peptidoglycan, while PA is composed of three lysin motifs (Lysm) derived from the C-terminus of the AcmA protein, capable of non-covalently binding to GEM. By fusing the ASFV antigens with PA, the ASFV antigens can be firmly attached to the surface of GEM. Subsequently, the piglets were immunized via intramuscular injection with a mixture of BLPs-F317L, BLPs-H171R, BLPs-D117L, BLPs-B602L, and BLPs-p54. Results: The results showed that the piglets developed detectable serum IgG antibodies 2 weeks after the first immunization, and these high antibody levels were maintained 4 weeks after the booster immunization. Moreover, these piglets produced more IFN-*γ*-producing lymphocytes than the control piglets. Conclusions: The data indicate that the generated BLPs mixture can stimulate both humoral and cellular immune responses in piglets, these five ASFV proteins are promising antigens, and the BLPs generated represent candidate ASF vaccines.

## 1. Introduction

African swine fever (ASF) is a highly contagious and hemorrhagic disease caused by African swine fever virus (ASFV) [1]. The clinical symptoms of ASFV-infected domestic pigs can vary from peracute and acute to subacute and chronic forms. Highly virulent ASFV strains typically induce severe clinical signs in pigs, with a mortality rate that can reach 100% [2,3]. While moderate and low virulent ASFV strains may not necessarily cause the death of all infected pigs, they can still result in prolonged virus shedding [4,5]. Apart from two vaccines licensed in Vietnam, no ASF vaccines are commercially available globally [6]. Thus, control measures rely solely on early diagnosis and stringent biosecurity measures, posing a significant threat to the global pig industry and related sectors. Therefore, developing safe and effective ASF vaccines is of utmost priority.

ASFV, a member of the *Asfarviridae* family, is a double-stranded DNA virus [7]. Its genome spans a size range of 170 to 194 kilobase pairs (kb) and encompasses 151 to 167 open reading frames (ORFs) [8]. ASFV encodes various structural and non-structural proteins that are essential for virus assembly, replication, repair, and viral genome expression [9,10,11]. However, the functions of some proteins remain unclear. Though live attenuated ASF vaccines have shown desirable efficacy in most cases, potential side effects and other safety concerns cannot be ignored [12,13]. Subunit vaccines against ASF, on the other hand, offer higher safety compared with live attenuated ASF vaccines. However, in clinical studies, ASF subunit vaccines have shown limited success [14,15]. This limited success may be primarily due to the selection of delivery systems and protective ASFV antigens.

The Gram-positive enhancer matrix-protein anchor (GEM-PA) is a novel exogenous protein display system. Specifically, this system utilizes *Lactococcus lactis* subjected to heat and acid treatment, during which intracellular components, including nucleic acids and all the cell wall components except peptidoglycan, are disrupted, resulting in hollow peptidoglycan particles that retain the bacterial cell shape [16]. PA is composed of three copies of the lysin motifs (Lysm) derived from the C-terminus of the AcmA protein, which is a peptidoglycan hydrolase in *L. lactis*. The Lysm enables non-covalent binding to peptidoglycan. Therefore, exogenous antigen protein can firmly bind to the surface of the GEM by fusing a PA. Since GEM only contains peptidoglycan, they can load exogenous proteins more effectively than living *L. lactis* [17]. Moreover, the glycan chains of peptidoglycan in the cell wall of *L. lactis*, cross-linked by short peptides and peptide bridges, can be recognized by toll-like receptors 2 (TLR2) [18]. This recognition subsequently induces pro-inflammatory cytokines, chemokines, and type I interferons (IFNs), and activates dendritic cells (DCs), promoting their maturation and enhancing antigen presentation efficiency, thus exhibiting good adjuvant effects [19,20]. Due to its robust capability to load exogenous proteins and their adjuvant properties, the GEM-PA system has gained attention in recent years as a promising vaccine carrier [21,22,23,24]. In this study, the GEM-PA display system was utilized to display five potential antigenic proteins (specifically, F317L, H171R, D117L, B602L, and p54), which were selected based on previous studies to evaluate their immunogenicity.

## 2. Materials and Methods

### 2.1. Plasmids and Bacterial Strains

The plasmid pGEX-6P-1-PA and the *L. lactis* NZ3900 strain were preserved in our laboratory. *Escherichia coli* DH5*α* and *E. coli* Rosetta were purchased from Sangon Biotech Co., Ltd. (Shanghai, China). The genome sequence of the ASFV HLJ/18 strain was retrieved from the GenBank database (accession number: MK333180.1).

### 2.2. Construction of the Prokaryotic Expression Vectors Expressing Fusion Proteins

Using the ASFV HLJ/18 strain genome as a template, specific primers containing homology arms of the pGEX-6P-1-PA plasmid were designed to amplify the target fragments of the *F317L*, *H171R*, *D117L*, *B602L*, and *E183L* (p54 protein gene) genes. To enhance protein expression levels, we utilized TMHMM-2.0 to predict the transmembrane regions of these antigens. Consequently, the amino acid sequences spanning positions 38-60 of the D117L protein and positions 30-52 of the p54 protein were deleted due to their location within transmembrane regions. Ultimately, the molecular weights of each protein were determined to be 37 kDa (F317L), 20 kDa (H171R), 18 kDa (D117L), 61 kDa (B602L), and 20 kDa (p54), respectively.

The specific primer sequences are listed in Table 1. The pGEX-6P-1-PA plasmid was double-digested with restriction enzymes *Eco*RI and *Bam*HI. According to the instructions of the ClonExpress II one-step cloning kit (Vazyme, Nanjing, China), the five ASFV genes (*F317L*, *H171R*, *D117L*, *B602L*, and *E183L*) were cloned in the pGEX-6P-1-PA vector to construct the recombinant plasmids. Next, 10 µL of the recombinant product was transformed into *E. coli* DH5*α* competent cells and cultured on Luria–Bertani (LB) agar plates containing 5% ampicillin. After overnight culturing at 37 °C, the plasmids were extracted for PCR verification. The plasmids with the correct size were sequenced for sequence alignment. Then, the correct plasmids were transformed into the expression host *E. coli* Rosetta to establish the prokaryotic expression system of the ASFV fusion proteins.

### 2.3. Prokaryotic Expression

The recombinant bacteria were incubated in 10 mL of LB medium containing ampicillin for induced expression. When the optical density at 600 nm (OD_600nm_) of the bacterial culture reached 0.3–0.4, 1 mM IPTG was added to the culture. After a 24 h induction for protein expression, the bacterial cells were collected and lysed by sonication to release proteins.

### 2.4. Western Blotting

A total of 40 µL of the lysed bacterial supernatant was mixed with 10 µL of 5× sodium dodecyl sulfate-polyacrylamide gel electrophoresis (SDS-PAGE) loading buffer and boiled for 10 min. The samples were then subjected to 10% SDS-PAGE and transferred onto a nitrocellulose membrane. Mouse anti-His tag antibody (diluted 1:1000) was used as the primary antibody, and goat anti-mouse IgG antibody (diluted 1:10,000) was used as the secondary antibody. The soluble expression of the fusion proteins was detected using a near-infrared scanner.

### 2.5. Preparation of the GEM

The *L. lactis* NZ3900 strain, stored in our laboratory at −80 °C, was streaked onto a GM17 agar plate. A single colony was picked and cultured overnight. Subsequently, the overnight culture was inoculated into 100 mL of GM17 medium and incubated at a 30 °C incubator for 12 h. The bacterial cells were collected by centrifugation at 10,000× *g* for 10 min. The pellets were washed three times with sterile phosphate-buffered saline (PBS) to remove the medium. The bacterial cells were then resuspended in 0.2 volumes of 10% trichloroacetic acid (TCA) and subjected to a boiling water bath for 30 min. The mixture was centrifuged again at 10,000× *g* for 10 min to remove the acid solution and washed five times with sterile PBS to remove the acid and impurities, including proteins and nucleic acids. The bacterial particles were counted using a hemocytometer, and the concentration was adjusted to 2.5 × 10^9^ particles/mL (defined as 1 U/mL). The cell concentration was then adjusted to 10 U/mL, and the GEM was stored at −80 °C for future use.

### 2.6. Binding of the Fusion Proteins to the GEM

The recombinant bacteria expressing the fusion proteins were induced with IPTG for 24 h. Subsequently, the bacterial cells were harvested by centrifugation and resuspended in 1 mL of sterile PBS. The cells were lysed through sonication, followed by the collection of the supernatants. A total of 1 U of the GEM was then added to the supernatant and incubated with gentle rotation at room temperature for 1 h. The mixture was centrifuged at 10,000× *g* for 10 min, and the resultant pellets were washed three times with sterile PBS. The final pellets containing the antigenic proteins of ASFV on its surface represented the prepared BLPs.

### 2.7. Immunofluorescence Assay (IFA)

The BLPs displaying the ASFV antigens on the surface were washed three times with PBS and centrifuged at 10,000× *g* for 5 min. The washed pellets were fixed in 1 mL of 4% paraformaldehyde and incubated with rotation at room temperature for 30 min. After fixation, the cells were washed three times with PBS. The fixed cells were then resuspended in 1 mL of 1% bovine serum albumin (BSA) and blocked at room temperature for 30 min. Following the blocking, the cells were washed three times with PBS. Subsequently, the cells were incubated overnight at 4 °C with rotation in 1 mL of reference anti-ASFV sera from the pigs that survived the infection with the *MGF300*-*2R*-deleted ASFV (1:200) [25]. After overnight incubation, the cells were washed five times with PBS. The cells were then incubated with 1 mL of FITC-labeled anti-pig fluorescent secondary antibody (diluted 1:200) at 37 °C in an incubator for 45 min. Following this, the cells were washed three times with PBS and resuspended in 200 µL of PBS. Ten microliters of the bacterial suspension were placed on a glass slide, covered with a cover slip, and allowed to dry naturally without bubbles. The fluorescence was observed using a laser-scanning confocal microscope.

### 2.8. Transmission Electron Microscopy Analysis

To observe the morphology of the BLPs-F317L, BLPs-H171R, BLPs-D117L, BLPs-B602L, and BLPs-p54, we used BLPs-p54 as a model to observe the *L. lactis* NZ3900, GEM, and BLPs-p54 by transmission electron microscopy (TEM). The specific steps were as follows: both samples were centrifuged at 10,000× *g* for 5 min. The resulting pellets were fixed with 2% glutaraldehyde for 24 h. After fixation, ultrathin sections were prepared and observed under a TEM.

### 2.9. Quantification of the Maximum Loading Capacity of the GEM

To determine the maximum binding capacity of 1 U of the GEM with each type of ASFV antigen protein, 200 mL of recombinant bacterial culture was induced and expressed. Subsequently, the bacterial cells were collected by centrifugation and resuspended in 20 mL of sterile PBS. The resuspended bacterial cells were then lysed by sonication, and the lysate was separated into supernatant and pellet. Different volumes (2, 3, 4, and 5 mL) of the sonicated supernatant were mixed with 1 U of the GEM and incubated at room temperature with rotation for 1 h. The mixture was washed three times with sterile PBS, with each wash involving centrifugation at 10,000× *g* for 5 min. The final pellets were resuspended in 1 mL of PBS to prepare the samples for analysis. SDS-PAGE was used to analyze the maximum binding capacity of 1 U of the GEM with the bacterial lysate. To establish a standard curve, various concentrations of BSA (0, 0.05, 0.10, 0.15, 0.20, 0.30, 0.40, and 0.50 mg/mL) were used as standard proteins. Equal volumes of these samples were subjected to SDS-PAGE analysis to generate a standard curve, which was then used to quantify the maximum binding amount of 1 U of the GEM with the ASFV antigens.

### 2.10. Animal Experiments

Eight 4-week-old commercial piglets negative for ASFV were obtained from the animal experimental base of the Harbin Veterinary Research Institute of the Chinese Academy of Agricultural Sciences. The piglets were randomly divided into three groups: group A consisted of 3 piglets, each received an intramuscular immunization of 5 U of the mixture of the BLPs (1 U each of the BLPs). This mixture was termed BLPs-ASFV-Mix, and the group was designated as the BLPs-ASFV-Mix group. Group B included 3 piglets, each immunized with 5 U of the GEM, and this is designated as the GEM group; group C comprised 2 piglets, each immunized with 2 mL of sterile PBS, and this group is defined as the PBS group. All piglets were immunized via intramuscular injection in the neck region. Each piglet underwent two immunizations, with a booster immunization administered 4 weeks after the initial immunization. Blood samples were collected at 2-week intervals post-immunization for further analysis. The detailed immunization schedule is outlined in Table 2.

### 2.11. Enzyme-Linked Immunosorbent Assay (ELISA)

The purified ASFV proteins (F317L, H171R, D117L, B602L, and p54) were diluted to appropriate concentrations in carbonate buffer solution and coated onto 96-well ELISA plates. The plates were then incubated overnight at 4 °C. Each well was washed three times with 200 µL of PBS containing 0.05% Tween 20 (PBST), and 100 µL of 5% non-fat dry milk was added as a blocking solution and incubated in a 37 °C incubator for 2 h. After removing the blocking solution, the plates were air-dried, and 100 µL of diluted serum samples (1:100) were added to each well, followed by incubation in a 37 °C incubator for 1 h. The plates were then washed five times with PBST for 3 min each time, and air-dried. Subsequently, 100 µL of diluted goat anti-pig IgG antibody was added to each well and incubated in a 37 °C incubator for 45 min. The plates were washed five times with PBST for 3 min each time. After the final wash, the plates were blotted on thick absorbent paper to dry. The tetramethylbenzidine (TMB) substrate solution (100 µL per well) was added, and the plates were incubated at room temperature in the dark for 15 min. The reaction was terminated by 50 µL of 2 M H_2_SO_4_ per well. Finally, the OD_450nm_ values were measured immediately.

### 2.12. Serum-Virus Neutralization Test

To measure the neutralizing activities of the sera from the piglets post-immunization against ASFV, a serum-virus neutralization test was conducted. Briefly, the porcine sera collected at 28 d post-booster immunization were inactivated in a 56 °C water bath for 30 min. The serum samples (SPF pig sera serving as a control) were then serially diluted using sterile PBS. An aliquot of 100 μL of the appropriately diluted serum samples was mixed with 200 TCID_50_/100 μL of rASFV-Gluc/EGFP, a recombinant dual-reporter virus constructed by our team. This virus did not affect virus replication or gene expression and allowed for the quantification of virus replication levels through Gluc activity. The virus–serum mixture was gently mixed and incubated at 37 °C in a 5% CO_2_ incubator for 1.5 h. Subsequently, the mixture was transferred onto porcine alveolar macrophages (PAMs) cultured in a 96-well plate and further incubated at 37 °C for 2 h. Afterward, the medium was replaced with fresh RPMI-1640 supplemented with 10% fetal bovine serum (FBS), and the cells were cultured at 37 °C in a 5% CO_2_ incubator for 72 h. Finally, 40 μL of the supernatant was collected to measure the Gluc activity, and the virus inhibition rates were calculated.

### 2.13. Enzyme-Linked Immunospot (ELIspot) Assay

The peripheral blood mononuclear cells (PBMCs) from the immunized piglets were isolated using a peripheral blood lymphocyte isolation kit. The test samples, negative and positive controls were assayed in duplicates concurrently. The wells were washed gently five times with PBS. RPMI 1640 medium containing 5% penicillin-streptomycin and 10% FBS (200 µL per well) was added, and the plates were incubated for 0.5 h. The medium was then discarded, and 100 µL of a cell suspension containing 5 × 10^5^ cells per well was seeded into the ELIspot 96-well plates. The test samples were stimulated with 100 µL of the ASFV HLJ/18 strain (10^6^ TCID_50_) per well. For comparison, an equivalent volume of RPMI 1640 medium served as a negative control, while phytohemagglutinin (PHA) diluted at 1:1000 was used as a positive control. Subsequently, the plates were incubated within an incubator maintained at 37 °C and 5% CO_2_ for 48 h. Following incubation, the culture medium was discarded, and the wells were washed five times with PBS. Then, 0.5 µg/mL of the detection antibody was added, and the plates were incubated at room temperature for 2 h. After removing the primary antibody, the wells were washed five times with PBS. A horseradish peroxidase (HRP)-labeled secondary antibody (diluted 1:1000 in PBS) was added and the plates were incubated at room temperature for 1 h. After removing the secondary antibody, the wells were gently washed five times with PBS. Next, 100 µL of TMB substrate solution was added to each well, and the plates were incubated in the dark for 30 min. The reaction was terminated by washing with water. After drying, the results were analyzed utilizing the AID iSpot FluoroSpot Reader System (ISpot Spectrum ELR088IFL; AID GmbH, Strassberg, Germany), and the number of the IFN-*γ*-producing T cells was calculated.

### 2.14. Flow Cytometry

About 10^6^ isolated pig PBMCs were immuostained with 2 µL of mouse anti-porcine CD3*ε*-SPRD (PPT3), mouse anti-porcine CD4-FITC (74-12-4), and mouse anti-porcine CD8*α*-PE (76-2-11) monoclonal antibodies (SouthernBiotech, Birmingham, AL, USA) on ice in the dark for 30 min. Single-staining cells with CD3*ε*-SPRD, CD4-FITC, or CD8*α*-PE were used as controls. The samples were then centrifuged at 500× *g* for 3 min at room temperature and the supernatants were discarded. The pellets were washed twice with 1 mL of PBS buffer and then resuspended in 500 µL of PBS buffer per tube. The samples were analyzed by flow cytometry to determine the proportion of CD8^+^ and CD4^+^ T cells per 10,000 cells, and the data were analyzed accordingly.

### 2.15. Data Analysis

The data were presented as the mean ± SD. Statistical analysis was performed using GraphPad Prism 9 (La Jolla, CA, USA). One-way ANOVA was used to determine the significance of differences between means. In the text, ns denotes *p* ≥ 0.05, indicating no significance; * denotes *p* < 0.05, ** denotes *p* < 0.01, *** denotes *p* < 0.001, and **** denotes *p* < 0.0001.

## 3. Results

### 3.1. The Five Fusion Proteins Could Be Expressed in Soluble Form

**Figure 1:**
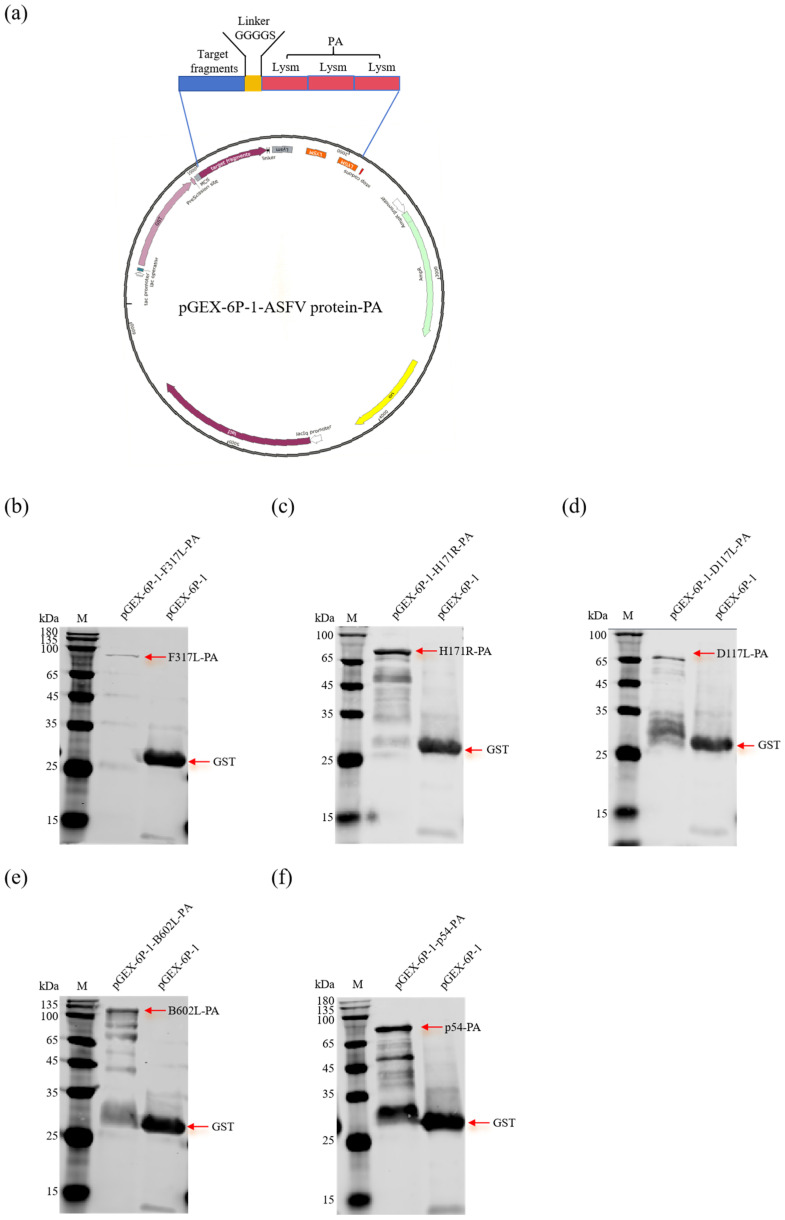
Construction of the prokaryotic expression plasmids and identification of soluble expression of the fusion proteins. (**a**) Schematic diagram of the recombinant plasmids expressing the fusion proteins. The plasmid pGEX-6P-1-PA was digested with *Eco*RI and *Bam*HI, and then the five ASFV genes (*F317L*, *H171R*, *D117L*, *B602L*, and *E183L*) were individually cloned in the pGEX-6P-1-PA vector to construct the corresponding recombinant plasmid. The ASFV proteins and the anchor protein PA were fused by a flexible linker (4GS). (**b**–**f**) Identification of the soluble expression of F317L-PA (**b**), H171R-PA (**c**), D117L-PA (**d**), B602L-PA (**e**), and p54-PA (**f**) by Western blotting. M represents the protein marker, and the red arrows represent the ASFV fusion proteins or the GST protein.

To enhance the soluble expression of the fusion proteins, we individually expressed five constructs in an *E. coli* prokaryotic expression system utilizing the pGEX-6P-1 vector. These constructs were designed by fusing each ASFV protein to the PA protein via flexible linkers (Figure 1a). To assess the level of soluble expression, we performed a Western blotting analysis on the supernatant of the bacterial lysate after sonication. The results revealed that all five fusion proteins F317L-PA (85 kDa), H171R-PA (69 kDa), D117L-PA (65 kDa), B602L-PA (110 kDa), and p54-PA (66 kDa) were successfully expressed in a soluble form within the bacterial cytoplasm, and the molecular weight of GST protein was approximately 26 kDa, while that of PA protein was approximately 21 kDa (Figure 1b–f).

### 3.2. The Fusion Proteins Could Be Anchored on the Surface of the GEM

**Figure 2:**
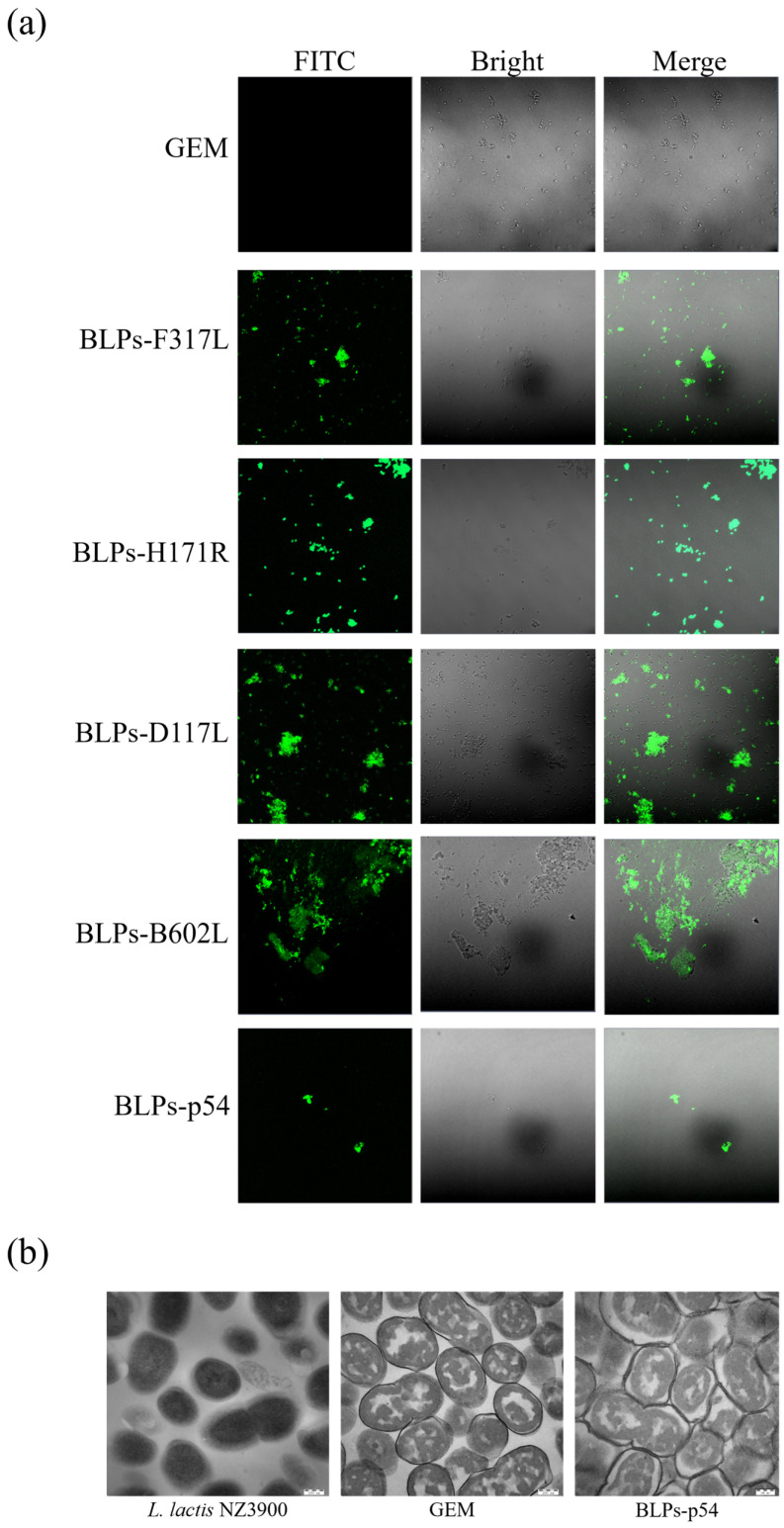
Identification of the surface display of the five ASFV antigens on BLPs and observation of the structure of BLPs. (**a**) Identification of the surface display of the five ASFV antigens on BLPs by IFA. Here, reference anti-ASFV sera were used as the primary antibody, and anti-pig IgG (whole molecule) FITC antibody was used as the secondary antibody. Subsequently, the samples were observed by a laser scanning confocal microscope. (**b**) TEM observation of *L. lactis* NZ3900, GEM, and BLPs-p54. The scale bars represent 200 nm.

To determine the anchoring position of the ASFV antigens on the GEM, the supernatant from the lysed bacteria, each containing the F317L-PA, H171R-PA, D117L-PA, B602L-PA, and p54-PA proteins, were individually assayed for binding to the GEM and observed by IFA. The results indicated that, after binding with the fusion proteins, the BLPs displayed specific fluorescence under confocal microscopy, whereas no specific fluorescence was observed in the GEM alone (Figure 2a). These results demonstrated that the ASFV antigens could be anchored and displayed on the surface of the GEM and specifically reacted with ASFV-positive serum. Since F317L-PA, H171R-PA, D117L-PA, B602L-PA, and p54-PA can all effectively anchor onto the surface of the GEM to form BLPs-F317L, BLPs-H171R, BLPs-D117L, BLPs-B602L, and BLPs-p54, we elected BLPs-p54 as a representative model for observing their morphologies. The BLPs-p54, GEM, and *L. lactis* NZ3900 cells were treated, and their structures were observed by TEM. The TEM images showed that the cell wall of the BLPs-p54 sample, after the GEM binding to the p54 fusion protein, was no longer smooth, with flocculent or punctate precipitates surrounding it. The GEM exhibited a lower electron density, with some contents leaked, but the bacterial skeleton and overall morphology were preserved. In contrast, the untreated *L. lactis* NZ3900 cells maintained their normal structure, with a darker cytoplasm and abundant contents (Figure 2b).

### 3.3. Maximum Loading Capacity of the GEM for the Exogenous Proteins

**Figure 3:**
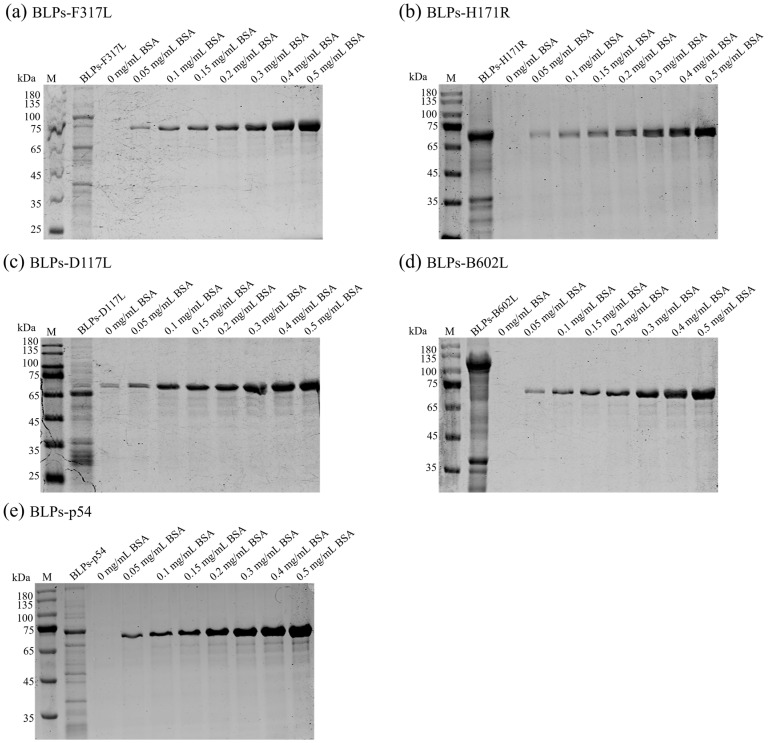
Quantification of the maximum loading capacity of the GEM. Determine the maximum loading capacity of F317L-PA (**a**), H171R-PA (**b**), D117L-PA (**c**), B602L-PA (**d**), and p54-PA (**e**) proteins for 1 U of the GEM by SDS-PAGE. Using different concentrations of BSA as a control, a standard curve was established to quantify the protein concentration of the sample. M represents the protein marker, and lanes 3 to 10 represent 0, 0.05, 0.1, 0.15, 0.2, 0.3, 0.4, and 0.5 mg/mL BSA standard protein, respectively.

To determine the maximum quantity of the recombinant proteins capable of binding to the GEM, 1 U of the GEM was incubated with 2, 3, 4, or 5 mL of the supernatants of the recombinant bacteria expressing the five ASFV antigens. After centrifugation, the pellets were analyzed by SDS-PAGE. The results showed that bands corresponding to the sizes of the five ASFV antigens were present in the GEM-bound samples, while no band was observed for the GEM alone, indicating that the GEM was free of contaminating proteins and could bind to the fusion proteins. The maximum binding capacity of 1 U of the GEM was determined to be 4 mL of sonicated recombinant bacterial supernatant (Figure S1). To further determine the loading capacity of 1 U of the GEM for each of the five ASFV antigens, we utilized BSA protein from a commercial BCA protein assay kit as a standard. The GEM was incubated with 4 mL of supernatant from recombinant bacteria expressing the fusion proteins, and the samples were analyzed using SDS-PAGE alongside different concentrations of BSA. The intensity of the bands was analyzed using the ImageJ software (Fiji 1.46r) to generate a standard curve and determine the maximum binding capacity of 1 U of the GEM for each ASFV antigen. The results indicated that, for 1 U of the GEM, the maximum loading capacities were 72 µg for F317L-PA, 350 µg for H171R-PA, 82 µg for D117L-PA, 152 µg for B602L-PA, and 61 µg for p54-PA, respectively (Figure 3).

### 3.4. BLPs-ASFV-Mix Induced High-Level Serum IgG in Piglets

**Figure 4:**
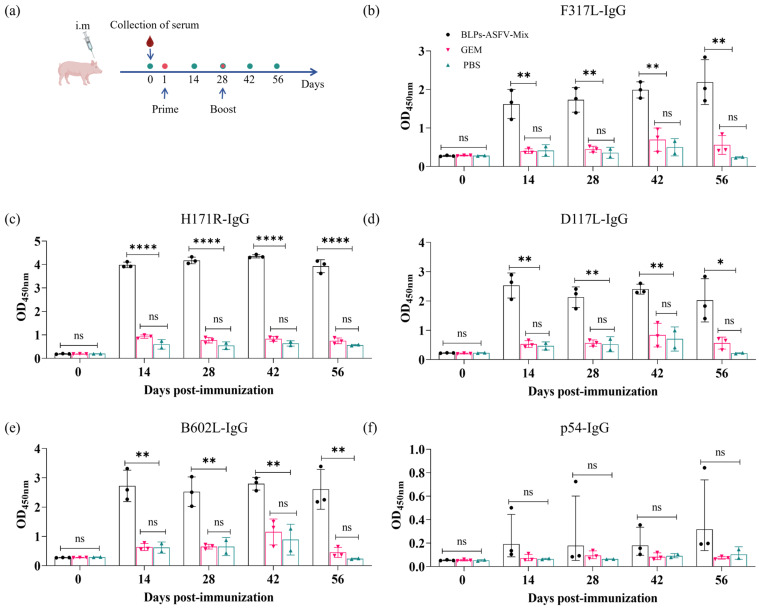
The serum ASFV antigen-specific IgG antibodies were produced in the immunized piglets. (**a**) Immunization schedule of piglets. (**b**–**f**) The level of serum ASFV antigen-specific IgG antibodies against F317L (**b**), H171R (**c**), D117L (**d**), B602L (**e**), and p54 (**f**) in the sera of the immunized piglets. Each ASFV antigen was used to coat 96-well ELISA plates, and the serum ASFV antigen-specific IgG antibodies against the different antigens were examined by ELISA. Statistical analysis was conducted using a one-way analysis of variance. Statistical significance was defined as the following: ns, *p* ≥ 0.05; *, *p* < 0.05, **, *p* < 0.01; ****, *p* < 0.0001.

To evaluate the immunogenicity of BLPs-F317L, BLPs-H171R, BLPs-D117L, BLPs-B602L, and BLPs-p54, three piglets in the BLPs-ASFV-Mix group were vaccinated intramuscularly (IM) with a mixture of these five BLPs. Three piglets in the GEM group were vaccinated with 5 U of the GEM by the IM route, while two pigs in the PBS group were administered 2 mL of PBS via the IM route (Figure 4a). After each immunization, serum samples were collected every 2 weeks to measure ASFV antigen-specific IgG antibody levels using an indirect ELISA. The results indicated that significantly higher-level IgG antibodies specific to the F317L, H171R, D117L, and B602L proteins were induced 14 d post-initial immunization in the BLPs-ASFV-Mix immunization group, and these high antibody levels were maintained for 4 weeks after the booster immunization. However, only one pig developed a notably high level of IgG antibodies specific to the p54 protein (Figure 4b–f). In addition, we conducted the serum-virus neutralization test to evaluate the neutralizing effects of the sera from the piglets immunized with BLPs-ASFV-Mix against ASFV. The results indicated that the sera from immunized piglets did not completely neutralize ASFV but exerted partial neutralization of ASFV. After co-incubation of 10-fold diluted sera with ASFV for 1.5 h, an inhibition rate of approximately 60% for ASFV was observed after three days. As the dilution multiple increased, the inhibitory effects on ASFV gradually weakened. But even at an 80-fold dilution, the inhibition rate remained approximately 38% (Figure S2).

### 3.5. BLPs-ASFV-Mix Induced Specific IFN-γ-Producing T Cells in Piglets

**Figure 5:**
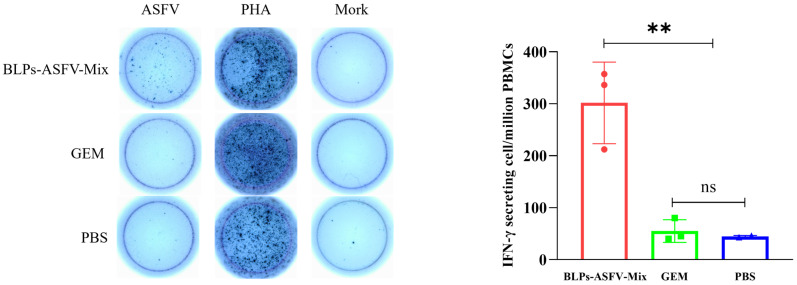
Quantification of antigen-specific IFN-*γ*-producing T cells per million PBMCs in the immunized piglets by ELIspot assay. The isolated PBMCs were stimulated with 100 µL of the ASFV HLJ/18 strain (10^6^ TCID_50_) per well. The results were read using the AID iSpot FluoroSpot reader system, and the IFN-*γ*-producing T cells per million PBMCs were calculated. The differences in the results were analyzed by a one-way analysis of variance. Statistical significance was defined as follows: ns, *p* ≥ 0.05; **, *p* < 0.01.

To evaluate the cellular immune response induced in the immunized piglets, we collected anticoagulation from the piglets 4 weeks after the second vaccination, isolated the PBMCs, and used ELIspot assays to quantify the number of IFN-*γ*-producing T cells in the peripheral blood. The results indicated that the number of IFN-*γ*-producing T cells in the PBMCs of the piglets immunized with BLPs-ASFV-Mix via IM injection was significantly higher compared with the GEM and PBS groups. There was no significant difference between the GEM and the PBS groups (Figure 5). To evaluate the numbers of CD4^+^ and CD8^+^ T cells in the PBMCs of piglets after intramuscular injection with BLPs-ASFV-Mix, anticoagulated blood was collected from the piglets 4 weeks after the second vaccination. PBMCs were isolated and subsequent analysis was conducted using flow cytometry. The results indicated that the piglets in the BLPs-ASFV-Mix group showed higher level of CD4^+^ and CD8^+^ T cells than did the GEM and the PBS groups. However, this improvement did not show a significant statistical difference when compared with the GEM and the PBS groups (Figure S3). These results suggest that immunization with the five BLPs expressing the ASFV antigens has the potential to activate CD4^+^ and CD8^+^ T cells, thereby stimulating the antiviral immune responses.

## 4. Discussion

Using *L. lactis* as a vaccine delivery vector is a promising strategy, but live *L. lactis* carriers can present challenges such as low expression levels of exogenous proteins and potential biosafety concerns. BLPs, derived from *L. lactis* through heat and acid inactivation, possess a hollow peptidoglycan structure while retaining bacterial morphology. As a non-transgenic microorganism devoid of recombinant DNA, BLPs exhibit a high safety profile. Furthermore, since the cell wall of the treated strains consists solely of peptidoglycan, the efficiency of anchoring target proteins to the particle surface is significantly enhanced [26,27]. Therefore, BLPs are considered an excellent vaccine carrier, and related vaccine products have already entered clinical trials [28]. In this study, five ASFV protective antigens (F317L, H171R, D117L, B602L, and p54) were individually fused with the PA protein and expressed. These fusion proteins were subsequently anchored and displayed on the surface of BLPs. In vivo trials in swine demonstrated that intramuscular immunization with a mixture of these five BLPs, each displaying an ASFV antigen, can induce both humoral and cellular immune responses. This approach represents a promising strategy for the development of BLPs-vectored ASF vaccines.

Considering that the prokaryotic expression system offers advantages such as easy operation, low cost, a short protein expression cycle, and high yield [29], we chose to express these five ASFV fusion proteins using this system. To improve the soluble expression levels of these fusion proteins, we selected pGEX-6P-1, which contains the soluble protein GST tag, as the expression plasmid. Additionally, we used *E. coli* Rosetta (DE3) as the host strain due to its ability to efficiently express rare codons. The results showed that the soluble expression levels of the five fusion proteins were significantly improved. Furthermore, the five fusion proteins could be efficiently anchored on the surface of BLPs. Efficient loading of foreign proteins is considered crucial for the carrier vaccine to exert its optimal effects.

Humoral immunity level is an important indicator to evaluate the effectiveness of a vaccine. Although ASFV infection is considered to be unable to produce traditional neutralizing antibodies, the humoral immunity index remains a significant consideration [30,31,32]. Our study showed that intramuscular immunization of piglets with BLPs-ASFV-Mix induced high level of serum IgG antibodies after 2 weeks post-initial immunization, which persisted at high level for up to 8 weeks. This may be attributed to the high antigen protein loading capacity of BLPs and their adjuvant effect [33]. However, unfortunately, only one piglet developed a high level of antibodies against the p54 protein. A possible explanation is that the p54 protein is a membrane protein [34], and to enhance its expression, we truncated its transmembrane region. Additionally, prokaryotic expression may result in the lack of corresponding modifications, thereby affecting its structure and immunogenicity. Furthermore, low binding affinity between the p54 protein and GEM, as well as aggregation phenomena during BLPs purification, could also be contributing factors. Furthermore, it has been shown that ASFV-positive sera can partially neutralize ASFV [35,36]. To evaluate the neutralizing efficacy of sera from the piglets immunized with BLPs-ASFV-Mix against ASFV, we employed a dual-reporter virus, rASFV-Gluc/EGFP, for neutralization test. The rASFV-Gluc/EGFP does not affect viral growth performance and protein expression, and the level of viral replication can be measured by determining the Gluc value. Consistent with previous reports, we found that even at a 10-fold dilution, the immunized sera confer partial neutralization of ASFV. This also demonstrates the potential of BLPs-ASFV-Mix as an ASF vaccine.

An increasing number of studies now acknowledge the crucial roles of cellular immunity in the prevention of ASFV infection. ELIspot can be used to quantitatively measure the number of cytokines secreted by individual cells. The number of IFN-*γ*-producing cells in the PBMCs of the immunized pigs can serve as an indicator of their antiviral cellular immunity level [37]. Our results showed that the number of IFN-*γ*-producing T cells in the piglets immunized with BLPs-ASFV-Mix was significantly higher than that in the GEM and the PBS groups. Subunit vaccines are typically known to produce low or no cellular immunity, necessitating the use of adjuvants to enhance their immune effect [38]. BLPs can promote the maturation of DCs and are recognized by TLR2, which in turn induces the production of pro-inflammatory cytokines, chemokines, and type I IFNs [39]. This mechanism may underpin the ability of the BLPs-ASFV-Mix immunization group to induce a discernible level of cellular immunity. Conceivably, the concurrent use of adjuvants that stimulate T cell immunity could further augment the cellular immune response elicited by BLPs-ASFV-Mix. To validate our previous observations, CD4^+^ and CD8^+^ T cells in the PBMCs of the immunized piglets were measured using flow cytometry. Specifically, the piglets immunized with BLPs-ASFV-Mix displayed an increasing trend in CD4^+^ T cells compared to both the GEM and the PBS groups, while CD8^+^ T cells also showed an increase compared with the PBS group, but there was no statistical difference compared with the control group of piglets. The number of immunized piglets may have contributed to this result. Increasing the number of immunized piglets in future studies could help improve the cellular immune data mediated by BLPs-ASFV-Mix.

However, it is worth noting that we still have many problems to be further improved and solved. One key challenge is to enhance the soluble expression levels of the ASFV antigens, while also ensuring its proper modification. Additionally, preventing the aggregation of BLPs may further improve its immunogenicity. The use of other adjuvants could also potentially boost the immunogenicity of BLPs. Most importantly, although the data indicate that BLPs mixture can induce both humoral and cellular immune responses in piglets, the evidence for the efficacy against ASFV infection in pigs is insufficient. Therefore, we need to conduct a challenge test to more accurately assess the protective potential of the BLPs-vectored ASF vaccine. In summary, despite these remaining challenges, our experiment still demonstrates that BLPs are a potential vaccine vector, and BLPs with surface display of the ASFV antigens represent a promising strategy for developing an ASF vector vaccine.

## 5. Conclusions

In this study, we explored the potential of the GEM as a vaccine vector for delivering the ASFV antigens. The results demonstrated that the five ASFV antigens could be anchored on the surface of the GEM, forming BLPs, and the data indicate that the generated BLPs mixture can stimulate both humoral and cellular immune responses in piglets. This indicates that the five ASFV proteins are promising antigens, and that the BLPs generated represent candidate ASF vaccines.

## Figures and Tables

**Table 1:** The primers for constructing the recombinant plasmids.

Names	Sequences (5′–3′)
F317L-PA-F	CACCAGGCGGCGGAGGTTCAATGGTTGAGACACAAATGGA
F317L-PA-R	CTATTGGATCCTCCGCCGCCTGTTGTGGACGATGCCTTTG
H171R-PA-F	CACCAGGCGGCGGAGGATCCATGGTAGTTTATGACTTGCT
H171R-PA-R	AATTCTGAACCTCCGCCGCCGTTTTTTATAGAAAACATGT
D117L-PA-F	CACCAGGCGGCGGAGGATCCATGGACACTGAAACGTCTCC
D117L-PA-R	AATTCTGAACCTCCGCCGCCTGAATGCGCAAGTTCAGCTA
B602L-PA-F	CACCAGGCGGCGGAGGATCCATGGCAGAATTTAATATTGA
B602L-PA-R	AATTCTGAACCTCCGCCGCCCAATTCTGCTTTTGTATATA
E183L-PA-F	CACCAGGCGGCGGAGGATCCATGGATTCTGAATTTTTTCA
E183L-PA-R	AATTCTGAACCTCCGCCGCCCAAGGAGTTTTCTAGGTCTT

**Table 2:** Immunization strategy in piglets.

Groups	Amounts	Immunogens	Doses
A	3	BLPs-ASFV-Mix	5 U/2 mL
B	3	GEM	5 U/2 mL
C	2	PBS	2 mL

## Data Availability

The dataset is available from the corresponding author upon reasonable request.

## Supplementary Materials

The following supporting information can be downloaded at: https://www.mdpi.com/article/10.3390/vaccines13010005/s1, Figure S1: The maximum binding amount of the GEM to lyse the supernatants of the recombinant strains expressing the ASFV antigens; Figure S2: The sera of piglets after immunization can significantly inhibit the replication of ASFV; Figure S3: The CD4^+^ and CD8^+^ T lymphocytes in the peripheral blood from the immunized piglets were analyzed by flow cytometry.
