# Supplementary material for: A Pool of Bacterium-like Particles Displaying African Swine Fever Virus Antigens Induces Both Humoral and Cellular Immune Responses in Pigs
Source: Vaccines (Basel). 2024 Dec 24;13(1):5. doi: 10.3390/vaccines13010005 (PMC11769380; doi:10.3390/vaccines13010005)
Supplement: Supplementary file 1 [file vaccines-13-00005-s001.zip › vaccines-3356592-supplementary.pdf]

Supplemental Figures

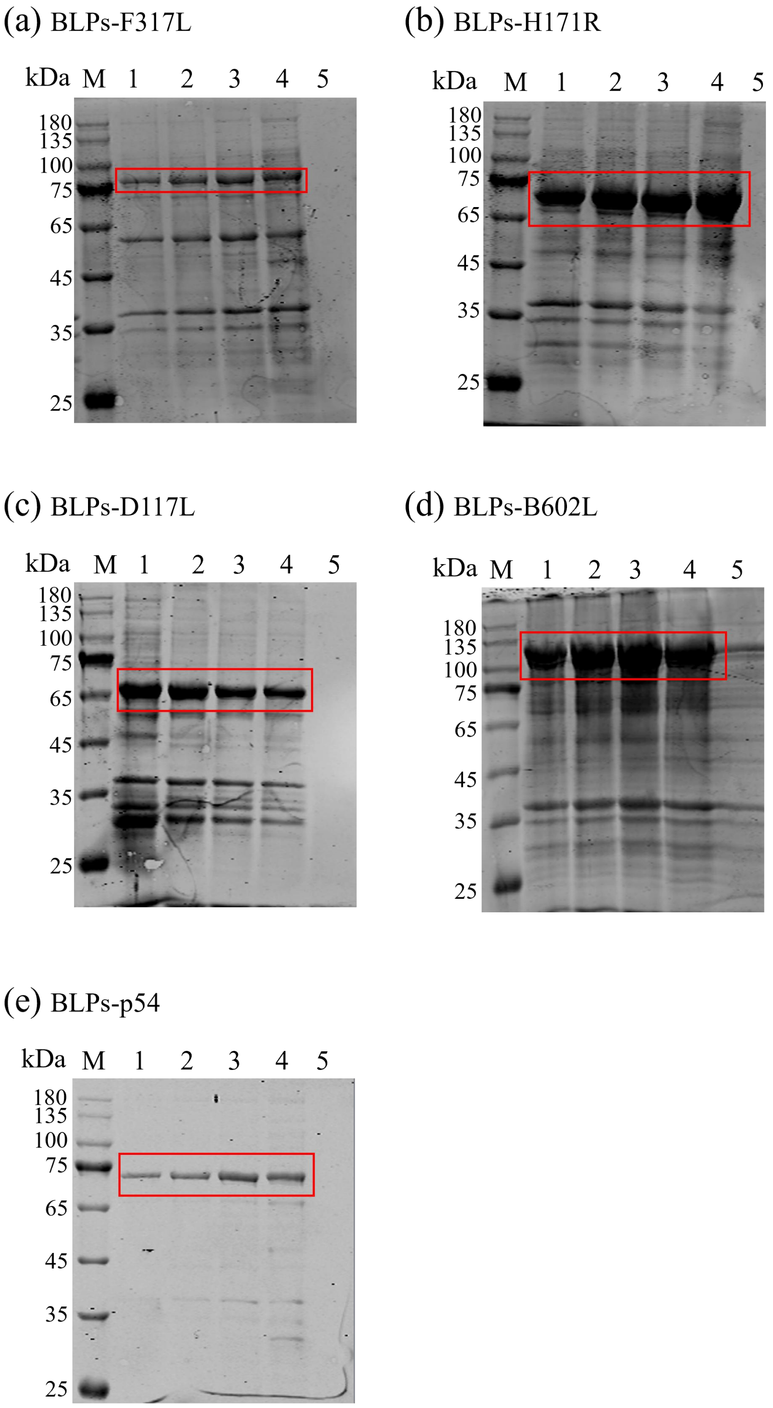

Figure S1. The maximum binding amount of GEM to lysis supernatant of recombinant strain expressing ASFV antigens. Determine the maximum binding amount of pGEX-6P-1-F317L-PA/Rosetta (a), pGEX-6P-1-H171R-PA/Rosetta (b), pGEX-6P-1-D117L-PA/Rosetta (c), pGEX-6P-1-B602L-PA/Rosetta (d), and pGEX-6P-1-p54-PA/Rosetta-PA (e) lysis supernatant for 1 U of the GEM by SDS-PAGE. M represents the protein marker, and lanes 1 to 4 represent 2, 3, 4, and 5 mL of recombinant strain lysis supernatant and 1 U of the GEM , respectively, and lane 5 represent 1 U of the GEM .

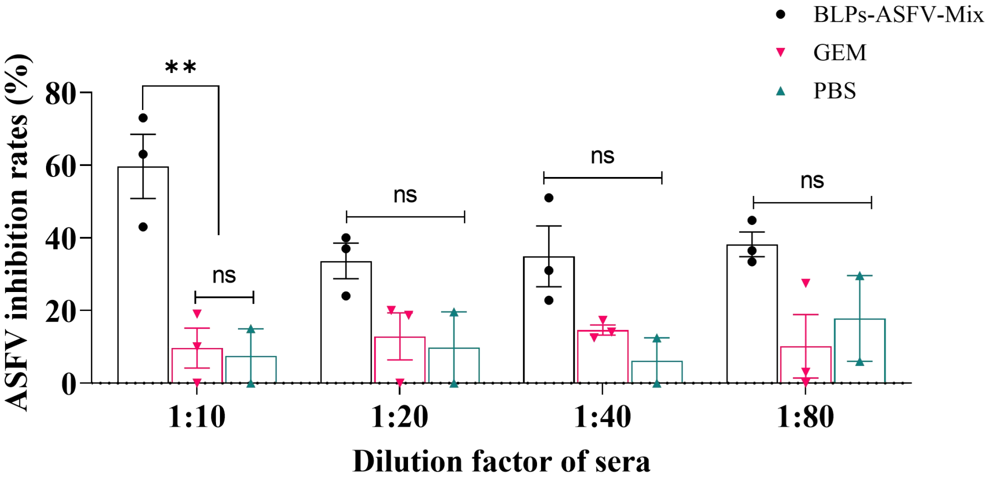

Figure S2. The sera of piglets after immunization can significantly inhibit the replication of ASFV. To measure the ability of post-immunization piglets sera to neutralize ASFV, a virus-neutralization test was conducted. The serum samples to be tested were serially diluted with sterile PBS and then co-incubated with 200 $TCID_{50}$/100μL of rASFV-Gluc/EGFP. The mixture was then used to infect PAMs. By calculating the GIuc value of cells in different groups, the inhibition rates were determined.

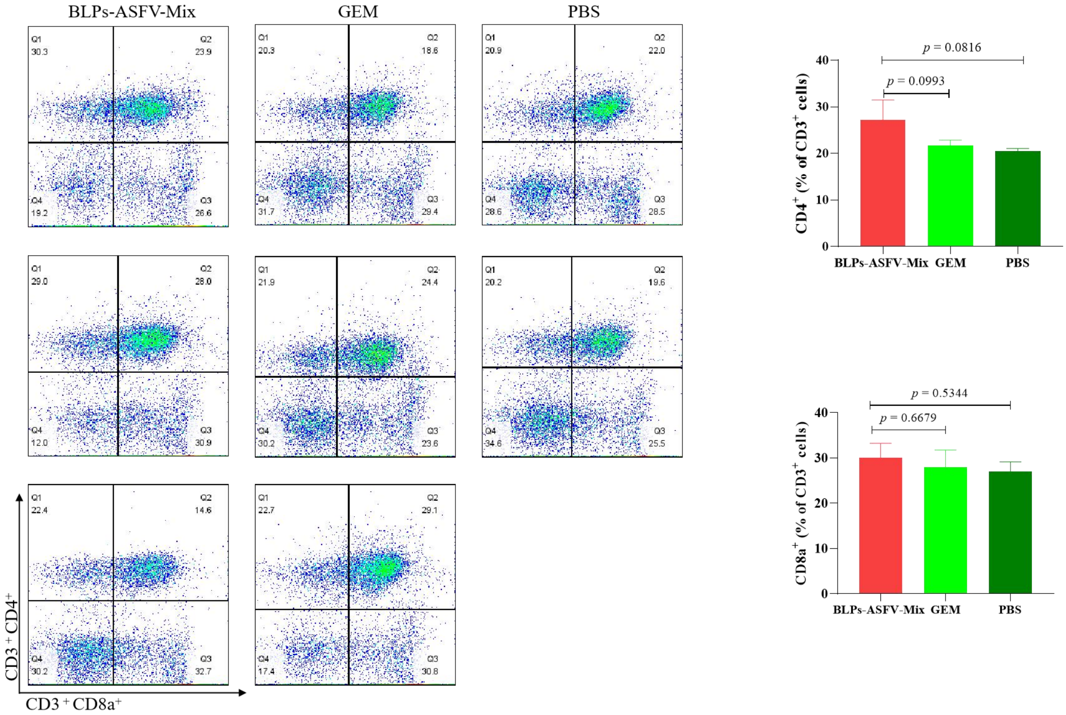

Figure S3. The $CD4^+$ and $CD8a^+$ lymphocytes in the peripheral blood from the immunized piglets were analyzed by flow cytometry. Single-staining cells with anti-CD3ε-SPRD, CD4-FITC, or CD8α-PE were used as a control, and when acquiring data on a flow cytometer, it is necessary to ensure that all cell populations to be analyzed fall within the visible range of the dot plot. If necessary, thresholds are set and adjusted to exclude most cell debris, bubbles, and laser noise interference from the analysis area. Flow cytometry was used to quantify the $CD8a^+$ and $CD4^+$ T cells per 10,000 cells.
